# Post Surgical Management of WHO Grade II Meningiomas: Our Experience, the Role of Gamma Knife and a Literature Review

**DOI:** 10.3390/life13010037

**Published:** 2022-12-23

**Authors:** Karol Migliorati, Giorgio Spatola, Lodoviga Giudice, Nine de Graaf, Chiara Bassetti, Cesare Giorgi, Marco Fontanella, Oscar Vivaldi, Mario Bignardi, Alberto Franzin

**Affiliations:** 1 Department of Neurosurgery, Fondazione Poliambulanza, 25124 Brescia, Italy; 2 Department of General Surgery, Fondazione Poliambulanza, 25124 Brescia, Italy; 3 Cancer Center Amsterdam, Department of Surgery, University of Amsterdam, 1012 WX Amsterdam, The Netherlands; 4 Medical Physics Unit, Fondazione Poliambulanza, 25124 Brescia, Italy; 5 Division of Neurosurgery, Department of Surgical Specialties, Radiological Sciences and Public Health, University of Brescia, 25124 Brescia, Italy; 6 Department of Radiation Oncology, Fondazione Poliambulanza, 25124 Brescia, Italy

**Keywords:** atypical meningiomas, WHO grade II meningiomas, Gamma Knife, radiosurgery, GKRS, Simpson grade

## Abstract

*Purpose:* Grade II meningiomas are rarer than Grade I, and when operated on, bear a higher risk of local recurrence, with a 5-year progression free survival (PFS) ranging from 59 to 90%. Radiotherapy (RT) or radiosurgery, such as Gamma Knife radiosurgery (GKRS) can reduce the risk of relapse in patients with residual disease, even if their role, particularly after gross total resection (GTR), is still under debate. Main goal of this study was to compare the outcomes of different post-surgical management of grade II meningiomas, grouped by degree of surgical removal (Simpson Grade); next in order we wanted to define the role of GKRS for the treatment of residual disease or relapse. *Methods:* from November 2016 to November 2020 all patients harboring grade II meningiomas, were divided into three groups, based on post-surgical management: (1) wait and see, (2) conventional adjuvant radiotherapy and (3) stereotactic GKRS radiosurgery. Relapse rate and PFS were registered at the time of last follow up and results were classified as stable, recurrence next to or distant from the surgical cavity. In the second part of the study we collected data of all patients who underwent GKRS in our Centers from November 2017 to November 2020. *Results:* A total of 37 patients were recruited, including seven patients with multiple meningiomas. Out of 47 meningiomas, 33 (70.2%) were followed with a wait and see strategy, six (12.7%) were treated with adjuvant radiotherapy, and 8 patients (17.0%) with adjuvant GKRS. Follow up data were available for 43 (91.4%) meningiomas. Within the wait and see group, recurrence rates differed based on Simpson grades, lower recurrence rates being observed in three Simpson I cases (30%) compared to twelve relapses (60%) in patients with Simpson grade II/III. Finally, out of the 24 meningiomas undergoing GKRS (8 residual and 16 recurrence), 21 remained stable at follow up. *Conclusions*: Gross total resection (GTR) Simpson II and III have a significantly worse outcome as compared to Simpson I. The absence of adjuvant treatment leads to significant worsening of the disease progression curve. Adjuvant radiotherapy, especially GKRS, provides good local control of the disease and should be considered as an adjuvant treatment in all cases where Simpson I resection is not possible.

## 1. Background

WHO Grade II meningiomas are rare and behave more aggressively when compared to WHO grade I meningiomas, with a higher tendency to local recurrence after surgery. Surgical resection is the treatment of choice for WHO grade II meningiomas and its extent is considered the main prognostic factor for recurrence [1,2]. Earlier reports showed a 5-year disease-free progression rate ranging from 59 to 90% after gross total resection (GTR), compared to 30 to 70% with sub-total removal (STR).

Postoperative treatment for meningiomas is determined by the extent of the surgical resection. The current classification system used to indicate the extent of surgical resection (Simpson grading) includes grades I to III for GTR, while grades IV–V are defined subtotal resection (STR) [3]. GTR of skull base meningiomas, is difficult to reach without important neurological sequelae. In Grade I GTR meningiomas no adjuvant treatment is recommended, while anaplastic meningiomas (grade III) require adjuvant radiotherapy (RT) [1]. In grade II meningiomas, the appropriate postoperative treatment strategy is still to be defined [4,5]. Current guidelines state that RT is indicated in patients with residual disease to reduce the risk of relapse [6,7]. The role of early adjuvant radiotherapy in patients with GTR has not been defined yet, and the options for radiotherapy or active monitoring are currently discussed on a case-by-case basis, according to the patient’s clinical status. The European study, ROAM/EORTC 1308, aimed to assess the best standard of care for patients with grade II meningioma undergoing GTR, and stated that, although adjuvant RT can avoid the need for further surgical procedures, its use must be weighed against the potential risks of side effects (3.4% to 16.7%), such as neuro-cognitive impairment, neurological damage (mainly of the visual pathways), hypopituitarism and radio-induced tumors [8]. 

Gamma Knife radiosurgery (GKRS), unlike standard RT, is a technique that selectively concentrates ionizing radiation on limited volumes, thus limiting side effects related to irradiation of large volumes [7]. As in surgery, grade II meningiomas respond differently to GKRS compared to grade I meningiomas and most of the studies refer to meningiomas with STR or relapses. 

This study analyzes and compares outcomes of different post-surgical management of grade II meningiomas with GTR in order to evaluate the role of adjuvant GKRS radiosurgery for the treatment of residual disease or relapse. 

## 2. Materials and Methods

This retrospective study was conducted at the Neurosurgery Unit of Fondazione Poliambulanza of Brescia in cooperation with the Neurosurgery Unit of Spedali Civili of Brescia.

### 2.1. Inclusion Criteria

From November 2016 to November 2020 all patients undergoing surgery in these two centers with histological diagnosis of grade II meningioma according to the latest WHO classification (2016), were included. We have chosen to retrospectively include only meningiomas from 2016 on, since the last WHO classification declared brain invasion sufficient to diagnose grade II, unlike previous classifications. All patients meeting the inclusion criteria were required to sign the informed consent to join the retrospective study and data collection. This retrospective study was approved by the institutional review boards. 

### 2.2. Exclusion Criteria

Patients with:

(a) Age under 16 years
(b) Type II neurofibromatosis (NF-2)
(c) Sheath meningiomas of the optic nerve
(d) Pregnant or breastfeeding women

### 2.3. Data Collection and Definitions

#### 2.3.1. Baseline Characteristics

Demographic, clinical and radiological data were collected. Additionally, information regarding the clinical symptoms of onset, the site of the meningioma, the possible presence of perilesional edema, the Karnofsky Performance Status (KPS) at the time of surgery, date of surgical procedure and degree of surgical excision were collected. Resection extent was classified using Simpson criteria and was based upon the neurosurgeons’ assessment and post-operative magnetic resonance imaging (MRI) findings. Gross totally resected tumors included Simpson grades I–III. Last, we have analyzed anatomopathological data, in particular: number of mitoses in high magnification fields, mitotic proliferation index and possible presence of cerebral invasion. 

#### 2.3.2. Post-Surgical Management

Age of patient, clinical post operative status, volume of the residue, mitosis index as well as the site of the meningioma were considered in our multidisciplinary board meetings. Radiologists, neurosurgeons and radiation oncologists were involved in deciding further adjuvant treatment. The absence of current guidelines for adjuvant treatments of grade II meningiomas, particularly after GTR, prompted us to decide case by case. Various clinical features of patients as well as individual criteria of surgeons and radiation oncologists influenced the decisions. 

As a result, patients were divided into three post-surgical management groups:

- Wait and see;
- Conventional adjuvant radiotherapy;
- Stereotactic GKRS radiosurgery.

For patients with STR (Simpson grade IV and V) the decision to carry out conventional radiotherapy instead of GKRS was based on residual volume, location, and the proximity to eloquent areas, always taking into consideration the neurosurgeon’s and the radiation oncologist’s criteria.

#### 2.3.3. Follow-Up

MRI sequences chosen were: multiplanar pre and post-contrast T1, T2, and FLAIR images. Post-surgery radiological assessment was obtained every six months. In case of recurrent or progressive meningioma, MRI documentation of recurrence or progression was required. Progression assessment was done by the enrolling institutions. 

Based on MRI imaging at follow up meningiomas were defined:

- Stable;
- Local/marginal recurrence: recurrence within 2 cm from the margins of surgical cavity;
- Distant recurrence.

For patients with relapse, the time to re-presentation of the disease (PFS), the volume at relapse and treatment modality (RT or GKRS) on relapse were collected. The minimum follow-up was 2 years (patients enrolled until 2020).

#### 2.3.4. Patient with GKRS Treatment

We have included all grade II meningiomas treated from 2017 (year of Gamma Knife acquisition in our Center) to 2020, regardless of whether they were residual or recurring, including surgery performed years before including cases from other centers. Data including initial treatment volumes, dose administered (including average, maximal and minimal dose), degree of coverage of the lesion, presence of any fractional treatment and onset of any side effects (which included increased cerebral edema, the presence of radionecrosis or the onset of post-treatment neurological deficits) were collected for all patients who underwent GKRS in our centers. Patient categories during follow-up were defined as stated above.

### 2.4. Statistical Analysis

A descriptive statistical analysis, regarding the demographic, clinical as well as radiological and histological characteristics of the patients was performed. Continuous variables are expressed as mean (SD) or median (interquartile range [IQR]) as appropriate, and categorical variables are presented as absolute numbers and percentages.

Kaplan–Meier analysis was used to estimate disease control (presence or absence of relapse) and progression-free survival (PFS), in relation to the degree of surgical removal and relative to the treatment received. Univariate analysis was performed using the log classification test. All factors with *p*-value ≤ 0.05 on univariate analysis were included in multivariate analysis using the Cox proportional hazards model. A *p*-value of ≤ 0.05 was considered statistically significant. Univariate analysis was performed using the log classification test also for patients undergoing GKRS, in order to understand if dose and volume were correlated with the response to radiosurgical treatment.

## 3. Results

Overall, we identified 37 patients diagnosed with grade II meningiomas from 2016 to 2020 in our centers. Table 1 shows the demographic, clinical and radiological characteristics of our series. Of the included patients,18 were male (48.6%). Mean age was found to be 66.79 years (range 37–82). Seven patients had multiple meningiomas for which a total of 47 meningiomas were included. 

Out of the whole group, three meningiomas were incidental findings, eleven presented non-specific symptoms (headache, ideo-motor slowdown, etc.), eight manifested epileptic seizures, seventeen had focal symptoms (hemiparesis, dysesthesia, visual disturbances, balance disorders), two patients started with behavioral alterations (disinhibition, alteration of character or mood) and one patient had the appearance of skin swelling as presenting symptom. All surgical cases were performed with the aid of navigation, except skull base meningiomas. Nine out of eleven meningiomas GTR (Simpson grade I) were located at the convexity, and two were falcine meningiomas. Out of the eleven meningiomas with STR (Simpson IV and V), three were located at the clinoid/cavernous sinus, three on the sphenoid wing, two parasagittal, two of the falx, two tentorial and one of the olfactory groove.

### 3.1. Post-Surgical Management

**Figure 1:**
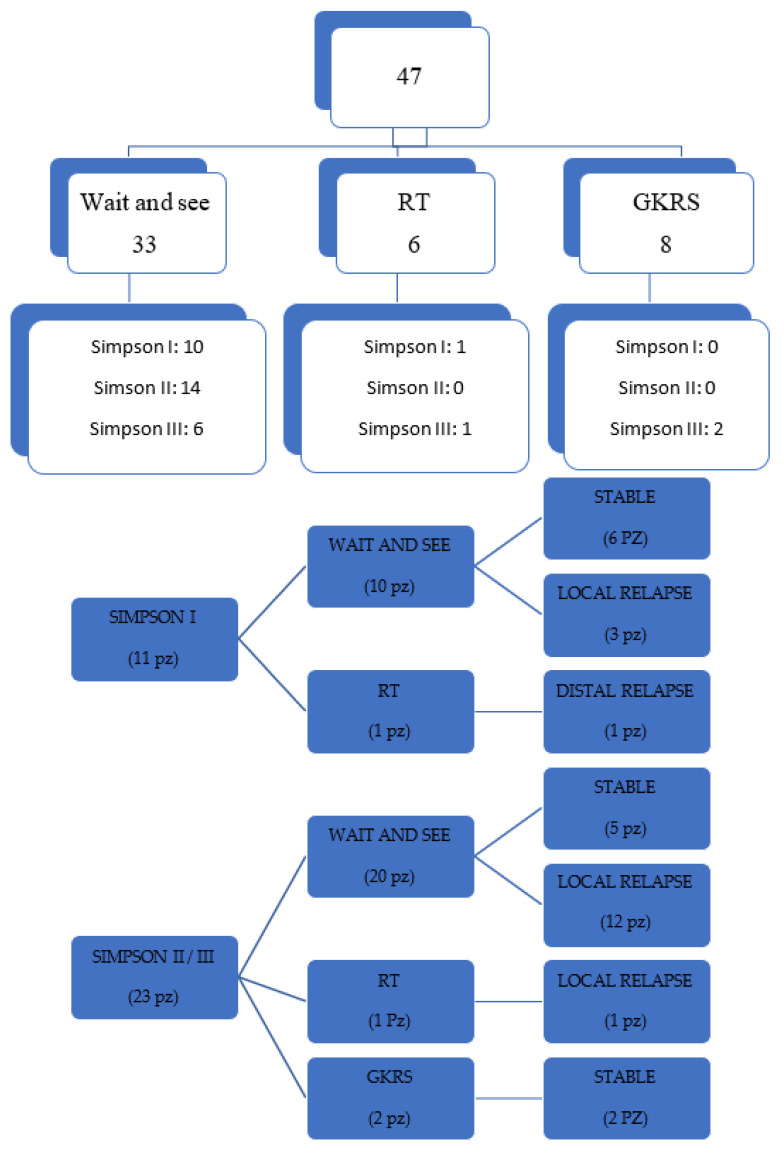
Illustrative diagram of postoperative management for all meningiomas (first picture) and follow up of patients with only gross total resection (GTR), stratified by Simpson grade (second picture). It does not include GKRS treatments on relapse.

The distribution of the different post-operative management according to the degree of surgical excision is shown in Figure 1. Of the 47 meningiomas analyzed, 33 (70.21%) underwent radiological monitoring, according to the wait and see strategy, 6 (12.76%) underwent adjuvant radiotherapy treatment and 8 patients (17.02%) received adjuvant GKRS, upon relapse, within 5 months of the surgical procedure. Of the 24 patients with local recurrence, 6 underwent a second surgical procedure, one patient underwent standard RT, and 16 patients underwent GKRS radiosurgery.

### 3.2. Follow-Up

Four patients were lost at follow-up. Of the 43 meningiomas available for follow up, 17 (36.17%) were stable, 24 (51.06%) had focal/marginal recurrence and 2 (4.2%) relapsed distally.

Of the patients who underwent a wait and see management, 19 (41.9%) had a relapse at 2 years, while 11 (25.6%) were stable at follow-up. Seven of the patients undergoing adjuvant therapy (both RT and GKRS), relapsed (30.2%). Frequency of relapse in patients in the wait and see group was significantly higher (41.9%) than that of patients with postoperative adjuvant treatment (30.2%).

Figure 1 shows the follow up, stratified by Simpson classification and by type of treatment. 

**Figure 2:**
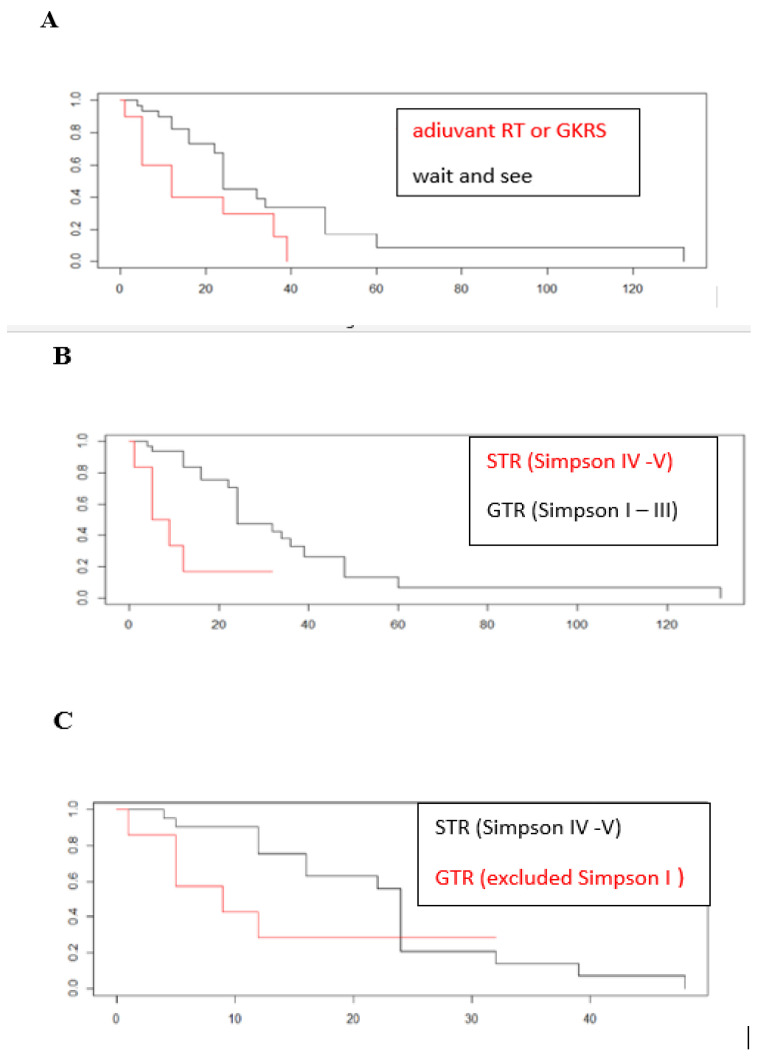
Kaplan–Meier for PFS (progression-free survival). PFS (months) is reported on the x axis, while on the y-axis shows the percentage of patients without relapse. In this case the event is represented by the presence of relapse. (**A**). Kaplan–Meier for patients undergoing wait and see (black line) and patients undergoing any adjuvant treatment (red line). Patients undergoing adjuvant treatment had a lower PFS than patients who received no treatment. This is explainable considering that the majority of patients subjected to a wait and see strategy had a GTR surgical removal while patients with adjuvant treatment only had an STR. (**B**). Kaplan–Meier progression-free survival for patients undergoing GTR (black line) and patients undergoing STR (red line). Partial surgical excision (Simpson IV–V), as represented by the univariate analysis, is the only significant risk factor for the presence of recurrence. (**C**) Kaplan–Meier progression-free survival for patients undergoing GTR—excluded Simpson grade I (red line) and patients undergoing STR (black line). In order to understand if PFS was greatly influenced by Simpson I patients, we deliberately excluded these patients from the curve and we saw that the PFS curve was reversed. This suggests that the Simpson II and III categories should be considered separately when assessing the likelihood of disease progression, especially with regard to decisions on possible adjuvant treatments.

The median PFS for patients who received postoperative treatment (RT or GKRS) was 12 months (range 0–39); for patients subjected to wait and see it was on average 17 months (range 4–132), as seen in Figure 2A.

The PFS related to the degree of surgical excision is shown in Figure 2B, while in Figure 2C the Kaplan–Maier curve was obtained excluding patients with Simpson I. In order to understand if PFS was greatly influenced by Simpson I patients, we deliberately excluded these patients from the curve and we saw that the PFS curve was reversed.

### 3.3. Simpson I

Data on the follow up stratified by Simpson grade are illustrated in Figure 1. Out of the 11 patients in Simpson I group, 10 underwent wait and see and one patient underwent adjuvant RT. The patient undergoing adjuvant RT showed, despite the diagnosis of meningioma grade II, some characteristics of anaplasticity, which is why, the collegial decision was to irradiate the surgical cavity. Six patients were stable, while three had local recurrence and one patient had distal recurrence.

### 3.4. Simpson II and III

Of the 23 patients with Simpson II and III removal, 20 went to the wait and see group, 12 of these relapsed locally, while 5 stayed stable. One patient underwent RT and two patients undergoing GKRS were stable at follow-up. 

Clinical and radiological parameters that may or may not influence the presence of relapse were identified using a univariate and multivariate analysis, as shown in Table 2.

The analysis shows that the only predictor of relapse is a degree of surgical removal higher than Simpson III (*p* = 0.0087). The risk of relapse in those who underwent total removal (Simpson I–III) is 0.25 compared to those with STR (Simpson IV–V). In other words the risk of recurrence in those who had Simpson I–III removal decreases by 75% compared to those whose tumor was partially removed (*p* = 0.0087).

### 3.5. Patients with GKRS Treatment

Table 3 shows the characteristics of patients treated with GKRS. A total of 19 patients were treated with GKRS, 3 with multiple meningiomas, involving a total of 24 meningiomas. In total, 8 patients received GKRS as an adjuvant treatment, while in the remaining 16 GKRS was carried out on relapse. Follow-up was available in 21 patients. Fractionated treatment was performed in 4 patients.

On univariate analysis, as shown in Table 4, no treatment GKRS parameter appears to be correlated with the probability of relapse. 

Of the three patients who relapsed, two had a subsequent histological diagnosis of anaplastic meningioma (WHO grade III) and are the same two patients in whom the relapse occurred distally. One of the two patients presented with mediastinal adenopathy, which later resulted in metastatic localization of the meningioma and died of cardiorespiratory arrest. The same patient had been a candidate for experimental oncocarbide therapy. In the third patient who died during follow-up there was a concomitant diagnosis of NSCLC, T3N2M1, stage IV lung adenocarcinoma and the patient died 4 months after GKRS due to pulmonary complications.

**Figure 3:**
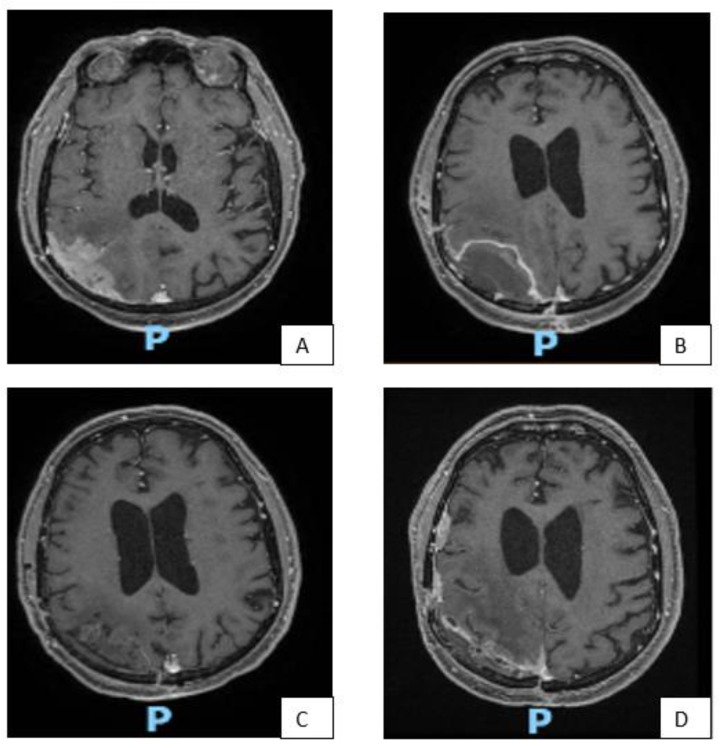

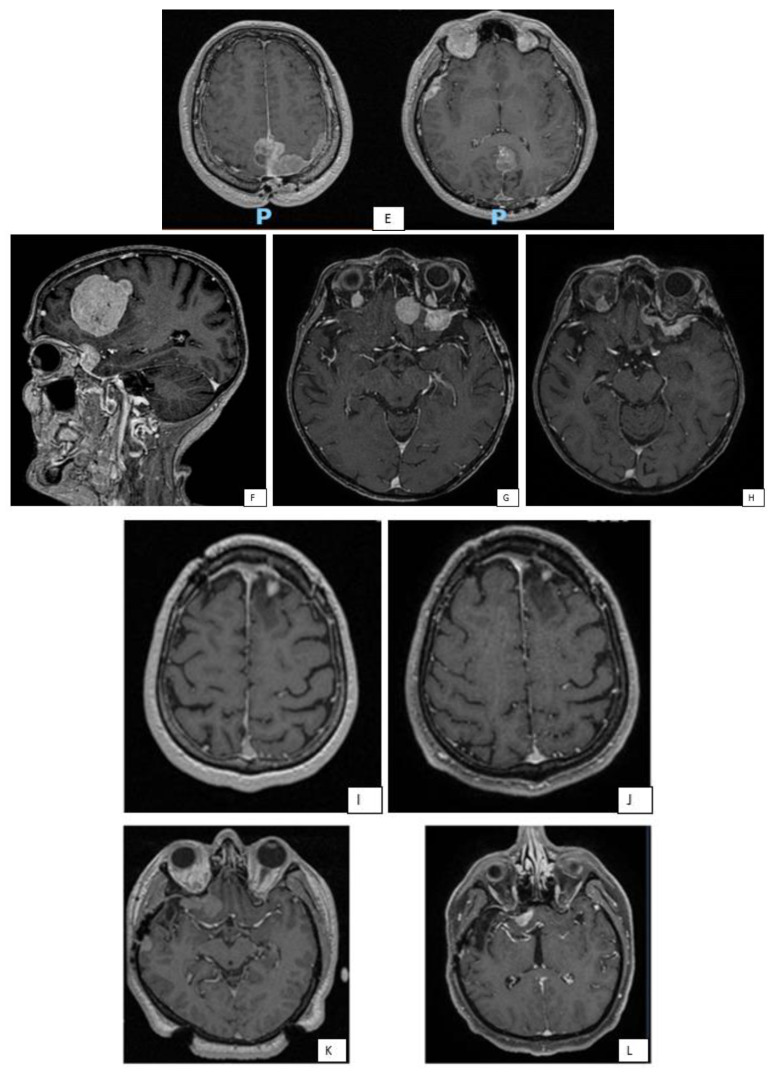
MRI images of some representative cases. Patient with right fronto-parietal convexity meningioma (**A**), subjected to GTR (Simpson I), as can be seen from the post-operative brain MRI (**B**). Due to the pathological features (high number of mitoses and cerebral invasion) the patient underwent adjuvant RT treatment despite surgical removal Simpson I. Good local control after two years from adjuvant RT (**C**). After 36 months, appearance of distal recurrence (**D**). MRI of a patient with multiple meningiomas (**E**). Patient with large left frontal meningioma, subjected to surgical removal (**F**), associated with two other nodules at the left sphenoidal wing, subjected to GKRS (**G**). Picture (**H**) shows good disease control 4 years after GKRS treatment. GKRS for a small recurrence of left frontal convexity meningioma (**I**) and relative MRI control 18 months after (**J**). GKRS for two nodules of right fronto-temporal meningioma (**K**). 15 months after radiosurgery, a new GKRS was performed on a further nodule of recurrence at the sphenoidal wing (**L**).

In Figure 3 some illustrative cases are reported.

## 4. Discussion

### 4.1. PFS and Simpson Grade

Data regarding WHO II meningiomas disease progression rates at 5 years after surgery range from 74% to 85% for Simpsons I and 34% to 89% in Simpsons II [9]. Although Simpson II and III are included in gross total resection (GTR), they are significantly different from Simpson I, where removal includes the base of implant. Some authors report no difference in PFS between Simpson grades I and II [10]. In our series, PFS of Simpson I without adjuvant treatment is 90% at 3 years, while PFS of patients with Simpson II and III is 45.5% and 37.5%, respectively. This suggests that Simpson II and III cases should be separately addressed with regard to decisions on possible adjuvant treatments. These results are in line with current guidelines, which recommend highest radical removal to achieve the best possible control over progression while underlining how the absence of adjuvant treatment may lead to a rapid relapse of the disease, in most cases within three years.

In our case series the high percentage of recurrence despite GTR, especially for Simpsons grade II and III in a short follow-up time span is relevant. Growing support is found in the literature for maximal safe resection as the primary goal in treatment of these patients, in an era of increasing availability of adjuvant treatment modalities [11]. Particularly in skull base meningiomas, Simpson I resection is difficult to achieve, Almost all meningiomas (81.81%) with Simpson grade I removal were convexity meningiomas, where removal of the dural base was surgically achievable, compared to meningiomas of the skull base or close to a venous sinus. Regarding postoperative management of GTR group, Figure 1 shows 30 meningiomas out of 34 (88.23%) undergoing a wait and see strategy, while only 2 receive RT treatment and 2 more GKRS radiosurgery. These results show that the two Centers involved favor an “observational” attitude in GTR of WHO II meningiomas, and this is in agreement with other clinical reports [4,5]. The debate on what treatment is best for grade II meningiomas radically removed, is still open [12]. Many studies report, after adjuvant treatment, a reduction in the relapse rate, an increase in PFS, as well as increased overall survival and better disease control in relapsing patients [13,14]. Other studies argue that adjuvant radiotherapy does not reduce the risk of disease recurrence and that damages and costs associated with radiotherapy are greater than the expected benefits [15,16]. A recent phase II study (RTOG 0539), examined the outcome of WHO II meningiomas with complete resection and adjuvant RT treatment using a standard dose of 54 Gy, reporting a 3-year PFS of 93.8%, significantly higher than controls and a relapse rate of 4.1% with low rates of toxicity induced by radiation [17]. Another phase II study (EORTC 22042-26042), using adjuvant RT treatment with higher doses (60 Gy) after complete surgical excision, reports a PFS of 90% at 3 years [18].

Currently there is an international, multicentric, randomized controlled, phase III study (ROAM-EORTC 1308), which aims to compare the outcome of patients with adjuvant RT with that of patients undergoing active monitoring [17].

In our series, the only patient in whom the adjuvant RT treatment was performed in a Simpson I, presented angiomatous aspects on histological examination with some microfocal areas of anaplastic change. This patient had local recurrence 36 months after surgery, despite RT of the surgical cavity, with important radionecrosis causing hemiparesis and medically poorly controlled epilepsy (Figure 3A–D). As shown in Figure 1 60% of patients with Simpson I surgical removal and observation, remained stable, while the percentage of stable in the Simpson II/III category fell to 25% (5 out of 20 patients). The only 2 patients with Simpson II/III treated with adjuvant GKRS, had stable follow up at 2 years.

The survival curves obtained considering the PFS of patients undergoing any adjuvant treatment (both RT and GKRS), compared with those that underwent wait and see strategy (Figure 2A) show that patients undergoing adjuvant treatment had a lower PFS than patients who received no treatment. This is due to the fact that the majority of patients not subjected to adjuvant treatment had a GTR while patients with adjuvant treatment only had an STR and therefore the best follow up depends on a “greater radicality” of surgery and not on the absence of adjuvant treatment as shown in Figure 2B. The univariate analysis confirms this observation, since the only parameter that correlates with the probability of recurrence appears to be the degree of surgical excision Simpson IV or V (*p* = 0.0087), with a recurrence risk rate of 0.25 in GTR (Simpson I–III) compared to STR (Simpson IV–V); in other words, the risk of recurrence in those who had a Simpson I–III removal drops by 75% compared to those who had partial removal performed (*p* = 0.0087). These data confirm what is found in the literature and what has already been indicated in the present guidelines: the most radical surgical removal possible must be pursued, without increasing perioperative morbidity and above all without increasing the risk of post-operative deficits (level of evidence III, recommendation level B) [7].

Finally, if patients with Simpson I are excluded from the analysis, as shown in Figure 2C, the survival curve is reversed, with a lower PFS in patients with Simpson II and III than in patients with Simpson IV and V. Considering again that most of the patients with Simpson II and III did not receive adjuvant treatment, this result confirms the high probability of relapse in this category, with a PFS that appears even lower than for patients undergoing STR but with adjuvant treatment. Therefore, if we exclude patients with Simspon grade I for grade II meningiomas with GTR Simpson II and III the absence of an adjuvant treatment leads to a significant worsening in the disease progression, with a PFS lower than that of meningiomas undergoing STR but with adjuvant radiotherapy.

### 4.2. GKRS Outcome

GKRS treatment represents a radiosurgery modality recommended in current guidelines, especially in WHO II meningioma residues, with local control reported at 2 years, ranging from 50% to 80% [19]. 

Table 5 represents the principal series who reported the results of GKRS for treatment of Grade II meningiomas. However, there are conflicting results on the effectiveness of radiosurgery in the treatment of aggressive meningiomas. In a recent paper timing of SRS after surgery seems to be of importance, with better results (longer PFS) reported when SRS is given as an adjuvant right after surgical resection, compared to when it is given as salvage treatment when a progression is observed, with a high incidence of distant tumor progression, despite a field tumor control of 84. They conclude that SRS is useful for the management of grade II and III meningiomas but does not cure them [20]. Nanda et al. examined 59 patients with grade II meningiomas of which 6 underwent GKRS on relapse. In their study, GKRS treatment did not achieve tumor control, as there was no significant difference in relapse-free survival with (52 months) or without GKRS (53 months, *p* = 0.41) [21]. In other reports the 5-year PFS rate after GKRS was 28%, lower than that reported in other studies; the authors explain this result as a consequence of a lower marginal dose (average of 14 Gy) due to, in many of the patients, previous radiotherapy or to large volume of the lesions treated [19]. In our series of 21 grade II meningiomas treated with GKRS available for follow up, there was good disease control (85.71%) at a mean follow up of 20–23 months with 18 stable patients (14.28%). Three patients relapsed, two with distal growth. For treatment of relapses of aggressive meningiomas, GKRS represents a good local control tool, but with poorer overall long-term control, due to the strong tendency of grade II meningiomas to invade and spread along the adjacent structures. Therefore, when analyzing the follow-up of patients undergoing GKRS, the main objective is the local control of the disease, since the possible presence of distant recurrence is more related to the intrinsic nature of the tumor rather than to a poor effectiveness of the method. Therefore, if we consider the local control rate of the tumor, defined as no evidence of local recurrence, the percentage rises from 85.71% to 95.23%, which seems satisfactory, despite the follow-up shorter than 3 years.

From many studies it emerges that dose, volume and timing of treatment after surgery are key elements in the outcome of patients [16,19,28,33,34]. From the univariate analysis of our case series, no correlation with the treatment parameters emerged, but this could be attributable to the small sample enrolled (Table 4).

Out of the two patients who relapsed after GKRS, two patients had a subsequent diagnosis of anaplastic meningioma. In the same two patients, there had been multiple treatments, both surgical and GKRS and the subsequent diagnosis of anaplastic meningioma is not certain whether it is attributable to a radio-induced transformation of the lesions or to an intrinsic advancement in tumor grading. One of the two patients also presented metastatic localizations in the thoracic level, leading to cardio-circulatory arrest. A previous history of radiation has already been associated by some studies with a worse outcome and a lower PFS [21]. The risk of malignant transformation with radiosurgery is a problem reported in the literature, with a frequency ranging from 18 to 27% [34]. Malignant transformation is thought to be due to an accumulation of genetic changes, such as loss of expression of CDKN2AB or mutations of the TERT promoter [34,35]. However, at the time of relapse it is rarely possible to have the opportunity for a second histological diagnosis. This could explain the phenomenon of some meningiomas with rapid growth or with a high tendency to relapse at a distance, from the resection site or from the area treated with GKRS.

In the third patient with marginal recurrence, there were two previous surgeries, respectively, in 2003 and 2014, with Simpson I removal in both procedures; the first GKRS treatment was performed 4 years after the second surgery, while the marginal recurrence after the first GKRS was 24 months later, and required a second radiosurgery treatment. The results obtained in this study regarding the local control of the disease are in line with the data in the literature, even if the follow-up in a few cases exceeds 3 years and this certainly represents a weakness of the current series. Additionally, for the four WHO grade II meningiomas with fractional GKRS treatment there was good disease control, with an average follow-up of 20.33 months. No adverse events due to radiosurgery treatment occurred in any of the patients analyzed.

### 4.3. Study Limitation

The retrospective study and the possible patient selection bias are the main limitation of this study, in addition to the smallness of the sample, which do not allow us to draw statistically significant conclusions; however these preliminary data are indicative of the high tendency to relapse of atypical WHO II meningiomas, even in patients with GTR but without removal of the base of implant. This should be taken more into consideration in the post operative management of patients with Simpson removal greater than grade I.

## 5. Conclusions

Despite the limited sample, in our series WHO II meningiomas with Simpson II and III resection show a significantly different outcome than those with GTR Simpson I, and the absence of adjuvant treatment may lead to a significant worsening in the disease progression curve, with a lower PFS than meningiomas undergoing STR but with adjuvant radiotherapy treatment. In our opinion, high incidence of recurrences in a short follow-up represents something to think about.

Stereotactic radiosurgery using GKRS provides good local control of the disease, both on residues and on possible relapses and should be considered as an adjuvant treatment in all cases where Simpson I excision is not possible, even if it does not prevent any distant relapse, typical of some more aggressive forms with anaplastic grading change.

## Figures and Tables

**Table 1:** Demographic, clinical and radiological characteristics of the patients analyzed.

	N° of Patients or Median (% or Range)
Age	66.79 (37–82)
Male	18 (48.6%)
KPS	
≥80	28 (68.3%)
<80	13 (31.7%)
Location	
Parasagittal	11 (23.4%)
Convexity	10 (21.27%)
Falx	10 (21.27%)
Lateral and middle sphenoid wing	7 (14.89)
Clinoidal e cavernous sinus	3 (6.38%)
Intraventricular	1 (2.12%)
Planum	2 (4.24%)
Tentorial	3 (6.38%)
Edema	
Yes	37 (76.6%)
No	11 (23.40%)
Simpson grade	
I	11 (23.4%)
II	14 (29.78%)
III	9 (19.14%)
IV	9 (19.14%)
V	4 (8.51%)
Neurological symptoms	
Incidental finding	3 (6.38%)
Headache	11 (23.4%)
Focal deficit	17 (36.17%)
Seizure	8 (17.02%)
Swellings	1 (2.12%)
Behavioral changes	2 (4.25%)
Anatomopathological features	
Ki67	15 (4–30)
N° mitosis	8.71 (4–20)
Brain invasion	12 (25.53%)
Follow up	
Present	43 (91.48%)
Months	28 (6–48)
Management post op	
Wait and see	33 (70.21%)
Adjuvant RT	6 (12.76%)
Gamma Knife	8 (17.02%)

**Table 2:** Prognostic factors of disease recurrence using univariate and multivariate analyzes. The only parameter that correlates with the probability of relapse after two years is GTR (Simpson I–III). The recurrence risk rate in those who have had total removal (Simpson I–III) is 0.25 compared to those who have had STR (Simpson IV–V); the risk of recurrence in those who have had Simpson surgical removal I–III decreases by 75% compared to those who carried out partial removal (*p* = 0.0087).

	Univariate Analysis		Multivariate Analysis	
	HR	*p* Value	HR	*p* Value
Age	1.01	0.66	1.035263	0.458939
KPS	0.999	0.99	0.942709	0.410907
Ki67	0.997	0.94	0.981073	0.778205
Mitosis	1.042	0.26	1.060144	0.444375
Brain Invasion	0.984	0.97	1.566417	0.217828
Simpson grade I–III (GTR)	*0.249*	0.0087	0.069585	0.026726
Edema	0.794	0.59	0.337299	0.212718

**Table 3:** Table of meningiomas treated by GKRS and the follow up. Only six patients underwent the GKRS as an adjuvant treatment of which only in two cases following a gross total resection (patient 1 and patient 2).

	Location	Simpson Grade	GK Adjuvant o Alla Relapse	Volume	Total Dose (Max/Min)	Fractionated GKRS	Follow Up (Months)	Complication
1, 58 y	falx	III	Adjuvant	10.916	13 Gy (9.2–26)	NO	Stable (24)	NO
2, 58 y	falx	III	Adjuvant	4.729	14 Gy (28–12)	NO	Stable (24)	NO
3, 75 y	covexity	II	Relapse (24 m)	6.594	15 Gy (30–10.9)	NO	Stable (22)	NO
4, 91 y	parasaggittal	III	Relapse (22 m) after RT	21.709	22.5 Gy (45–16.8)	SI	Stable (13)	NO
5, 71 y	falx	II	Relapse (16 m)	9.891	15 Gy (30–7.1)	NO	Stable (23)	NO
6, 80 y	falx	III	Relapse (12 m) + RT	24.411	15 Gy (24–7.3)	NO	Stable (25)	NO
7, 78 y	tubercle	IV	Adjuvant	0.305	14 Gy (28–12.4)	NO	Stable (15 m)	NO
8	tentorial	IV	Adjuvant	3.155	13 Gy (26–11.5)	NO	Stable (15 m)	NO
9, 80 y	covexity	I	Relapse (34 m)	1.633	15 (30–13.5)	NO	/	/
10, 59 y	Falx	III	Relapse (4 m)	9.25	25 (48.8–19.1)	SI	Distal relapse	┼
11, 73 y	Sphenoid ridge	III	Adjuvant	0.437	15 Gy (27.3–13.3)	NO	/	/
12, 73 y	falx	IV	Adjuvant	4.528	15 Gy (30–13.4)	NO	/	/
13, 49 y	Sphenoid ridge	II	Relapse (26 m) +RT	4.257	15 Gy (30–12.3)	NO	Stable	NO
14, 67 y	Falx	IV	relapse	2.607	15 Gy (12.9–28.0)	NO	Distal Relapse	┼
15, 71 y	Falx	III	Relapse (30 m)	4.651	15 Gy (30–12.6)	NO	Stable (18)	NO
16, 68 y	Parasaggittal	I	Relapse (48)	0.350	15 Gy (30–12.5)	NO	/	/
17, 67 y	Covexity	I	Relapse (48)	2.616	15 Gy (30.3–12.8)	NO	Local relapse	NO
18, 64 y	Tentorial	III	Relapse (24 m)	10.069	21 Gy (42–17.1)	SI	/	/
19, 63 y	Sphenoid ridge	II	Relapse (22 m)	2.567	15 Gy (30–10.6)	NO	Stable (6 m)	NO
20, 62 y 21	Covexity Covexity	I I	Relapse (12 m) Relapse (12 m)	5.355 0.357	15 Gy (30–11.3) 15 Gy (30–12.6)	NO NO	Stable stable	┼ (NSCLC)
22, 68 y	Falx	III	Relapse (21 m)	3.412	15 Gy (29.8–13.1)	NO	stable	NO
23, 74 y	Planum	IV	Adjuvant	2.239	25 Gy (50–20.4)	SI	Stable (24 m)	NO
24, 74 y	Sphenoid ridge	IV	Adjuvant	3.997	25 Gy (46.7–18.4)	SI	Stable (24 m)	NO

┼: dead patient.

**Table 4:** Prognostic factors of recurrence: none of the parameters analyzed relating to the Gamma Knife treatment (volume, mean dose, maximal and minimal dose) seems to correlate with the probability of recurrence (*p* ≥ 0.05).

	HR	*p* Value
Volume	0.76322	0.40663
Medium dose	3.34565	0.47343
Maximum dose	0.89888	0.40663
Minimum dose	0.4864	0.47343

**Table 5:** Main published series concerning the role of GKRS in the treatment of WHO grade II and III meningiomas.

Series	N Patients	N Tumors	Volume (cm^3^)	Marginal Dose (Gy)	Follow Up (Months)	PFS
Stafford [22]	22	22	8.2	16	40	68% at 5 y
Harris [23]	18	18	13.5	15	28	83% at 5 y
Huffmann [24]	15	21	5	16	6	93% at 6 months
Malik [25]	/	23	7.3	20	44	49% at 5 y
Kondziolka [26]	54	54	7.4	14	48	50% at 2 y
Attia [16]	24	24	7.9	14	43	25% at 5 y
Kim [19]	35	35	3.5	16	29	53% at 2 y
Pollock [27]	50	71	14.6	15	38	40% at 5 y
Hanakita [28]	22	28	6	18	23.5	20.4% at 5 y
Mori [29]	19	22	8.6	16.5	28	34% at 3 y
Tamura [30]	9	9	7.1	18.8	37	/
Ferraro [31]	31	31	3.9	18	34.5	70.1% at 3 y
Wang [32]	46	66	11.7	12.5	32.6	20.4% at 5 y
